# Miscellaneous

**Published:** 1888-06

**Authors:** 


### MISCELLANEOUS.

TRUTH STRANGER THAN FICTION.—Had a person who was living on earth ont hundred or even fifty years ago, bœen told facts that are transpiring to-day—facts that are common to-day—you could not have made him believe that such things could be possible. Even Franklin could not have foreseen what wonderful developments were about to take place in the uses of electricity. Had Fulton been told what advancement would be made in the power of steam, he would have said it cannot be. Had Daguerre been informed what great improvements were going to take place in the line to which his whole life was so deeply devoted, he would have doubted. And when we take even a casual glance at the great unfoldments that have taken place during the last fifty years, we feel almost appalled at its magnitude ; and still the world moves ; still new developments are being made on every hand, and who can foresee what will transpire on "the morrow ?"

When our much esteemed president, Capt. J. H. McMillen, was in Boston, last fall, he was told by the spirit of his first wife that if he would go to a certain place that she would be there and would sit for a portrait. He did as directed. The artist was an entire stranger to him, and he gave him no information either of himself or family, but simply said he came there for a portrait. In less than two hours, and in the presence of Capt. McMillen and his wife, the artist drew a crayon likeness of his former wife, who died in 1850. The picture is life size and is readily recognized by old acquaintances of the family. This was so fine that Capt. McMillen and his wife took five other sittings, each time getting fine crayon pictures of their children and members of their family. The first was a portrait of a son of Capt. McMillen, by his first wife, the mother dying when the child was only eight days old—the boy dying at twelve years of age; the third picture was of a son by his present wife, who died at the age of ten years. Then there came two other pictures of children by his present wife, and lastly, a portrait of Mrs. Barton, the mother of his present wife, and readily recognized by Mrs. McMillen as being a correct likeness of her mother. These pictures are a very fair work of art, all full sized, and when we take into consideration that only a little over one hour was devoted to such pictures, and the fact that the likenesses are all correct, and that the spirit through the artist, informed the parties who they were, and all aboue themselves, it becomes truly wonderful, and we are led to exclaim, what next ?—C. A. REED, in *Golden Gate.*

THE STERLING BAKING POWDER.—A trial of the Sterling Baking Powder is all that is necessary to convince the housewife that it is the article she has been looking for. It is a pure wholesome compound, entirely free from any deleterious ingredient, and has become the standard powder in the market.

DEDICATION OF PRESS HEADQUARTERS.—We have the pleasure to acknowledge the receipt of a very handsomely engraved card of invitation to the ceremonial "Dedication of the Press Headquarters," of the Cincinnati Press Club, at the Centennial Buildings, June 9, 1888, and regret our inability to be present on so interesting an occasion.

THEY FOUND A VICTIM.—Nellie Bryant, the pretty New Boston girl, is dead, a victim to the mind cure. Her distracted parents, once converts to the theory of Christian science, now curse the name, while the neighbors who loved and admired the beautiful girl, are both loud and deep in their denunciation of the quacks who parade under the name of Christian scientists.

" The girl died," said Dr. Ripley, last night, "of want of care, typhoid fever and Christian science."

Nellie Bryant was attacked some time ago with virulent typhoid fever. Mrs. Moore, a scientist, was given the case, and conducted it as usual with her peculiar creed. She gave no medicine, ordered no diet, and took none of the steps known to the profession, but she worked the mind cure. In the meantime the patient was allowed to eat such laxatives as watermelon, grapes, other fruit, and, indeed, what she pleased. The result was she grew rapidly worse. The neighbors saw how the case was tending, and some of them wrote to the city Board of Health, asking it to interfere and save the girl from certain death. This, of course, the Board could not do. The girl grew steadily worse, and finally her father, four days ago, dismissed Mrs. Moore and called in Dr. Martha Ripley, but it was too late. The sufferer was then beyond aid, and slowly sank until death relieved her.

Besides being a shock to the community, this will be a shock to the numerous credulous people who firmly believe in Christian science, but the awakening may do no harm.—*St. Paul Globe.*

### THE PARTING.

FROM THE GERMAN OF HEINRICH HEINE.

(Translated for HALL'S JOURNAL OF HEALTH.)

When two lovers fondly part,
Hand in hand and heart to heart,
Only for a season ;
Sighs will come and tears will start—
Know ye not the reason ?

We have parted, you and I,
With a passionless good-bye,
Every tie to sever ;
But the later tear and sigh
Mock the vain endeavor.

